# Increased CD4^+^ T Cell Co-Inhibitory Immune Receptor CEACAM1 in Neonatal Sepsis and Soluble-CEACAM1 in Meningococcal Sepsis: A Role in Sepsis-Associated Immune Suppression?

**DOI:** 10.1371/journal.pone.0068294

**Published:** 2013-07-22

**Authors:** Michiel van der Flier, Dyana B. Sharma, Silvia Estevão, Marieke Emonts, Denise Rook, Jan A. Hazelzet, Johannes B. van Goudoever, Nico G. Hartwig

**Affiliations:** 1 Department of Pediatric Infectious Diseases and Immunology, Erasmus MC - Sophia, Rotterdam, The Netherlands; 2 Laboratory of Pediatrics, Pediatric Infectious Diseases Group, Erasmus MC - Sophia, Rotterdam, The Netherlands; 3 Neonatal Intensive Care Unit, Erasmus MC - Sophia, Rotterdam, The Netherlands; 4 Pediatric Intensive Care Unit, Erasmus MC - Sophia, Rotterdam, The Netherlands; University of Cincinnati, United States of America

## Abstract

The co-inhibitory immune receptor carcinoembryonic antigen-related cell-adhesion molecule 1 (CEACAM1) and its self-ligand CEACAM1 can suppress T cell function. Suppression of T cell function in sepsis is well documented. Late-onset neonatal sepsis in VLBW-infants was associated with an increased percentage CEACAM1 positive CD4^+^ T-cells. Meningococcal septic shock in children was associated with increased serum soluble CEACAM1. In conclusion our data demonstrate increased surface expression of the co-inhibitory immune receptor CEACAM1 in late-onset neonatal sepsis in VLBW-infants, and increased circulating soluble CEACAM1 in children with meningococcal sepsis. Increased T-cell CEACAM1 expression and increased circulating soluble CEACAM1 may contribute to sepsis-associated immune suppression.

## Introduction

Sepsis is defined as a systemic inflammatory syndrome in response to an infection. Sepsis is an important cause of pediatric morbidity and mortality [1]. The host inflammatory response in sepsis is characterized by aspects of both a hyperactive immune response and immunosuppression [2]. Suppression of T-cell function and T-cell apoptosis in sepsis is well documented [2–4]. The mechanism of T-cell suppression is, however, not fully understood. Immune co-receptors on myeloid and lymphoid cells modulate the response of immune activating receptors and are crucial in regulating inflammation [5,6]. Recent data support an important role of costimulatory molecules in the regulation of inflammation in severe sepsis, and demonstrate an increase in the percentage CD4^+^ T-cells expressing the immune inhibitory receptor cytotoxic T lymphocyte antigen-4 (CTLA-4) [7,8].

 [5,6]The carcinoembryonic antigen-related cell-adhesion molecule 1 (CEACAM1) has recently been recognized as a regulatory co-receptor for both myeloid and lymphoid cell types [9]. Most studies have ascribed an inhibitory function to CEACAM1 in T-cells. Ligation of CEACAM1 on T cells induces a signal cascade that leads to suppression of T cell cytokine production and proliferation [10,11]. In vitro activation of T-cells by cytokines such as IL-2, IL-7 and IL-15 causes rapid and strong CEACAM1 up regulation, which persists for many days [12]. CEACAM1 is activated by its self-ligand CEACAM1.

We hypothesized upregulation of CEACAM1 occurs in sepsis. Firstly we tested whether CD4^+^ T-cell CEACAM1 expression is increased in very low birthweight (VLBW) infants (birth weight 401-1500 gram) with late-onset neonatal sepsis. Secondly, we tested whether serum soluble CEACAM1 concentration is increased in children with meningococcal septic shock. Our results demonstrate for the first time that CEACAM 1 is increased in sepsis.

## Methods

### Patients and controls

#### Ethics statement

The medical ethics committee of the Erasmus University Medical Center Rotterdam and University Medical Center Utrecht approved the study protocols and written informed consent was obtained from parents or legal representatives of children. For the use of surplus blood samples in control very-low birth weight infants verbal consent from parents or legal representatives of children was obtained. No written consent was deemed necessarily for the use of surplus blood samples by the ethics committee, all parents received written information on the use of surplus blood samples for research purposes and were asked if they agreed. The medical ethics committee of the Erasmus University Medical Center Rotterdam approved this procedure.

Two groups of pediatric sepsis patient were studied: very-low birthweight (VLBW) infants with late-onset neonatal sepsis and children with meningococcal sepsis. The reported studies have an exploratory nature, hence no power calculation was performed.

#### Neonatal sepsis sub study

In the neonatal sepsis group twelve very-low birthweight infants with late-onset neonatal sepsis were included between April and September 2008, at the Neonatal Intensive Care Unit (NICU) of the Sophia Children’s Hospital, Erasmus Medical Centre. Etiologic agents were *Escherichia coli* (n=2), coagulase negative staphylococcus (n=6), no pathogen isolated n=4). The group size of the late-onset sepsis group was determined by study enrollment of consecutive late-onset sepsis cases during the study period and arbitrary the minimal number of controls was set on 16.

Neonatal sepsis was defined as a bloodstream infection following a previously published definition for culture confirmed and clinical bloodstream infection in (premature) neonates [13]. For the diagnosis of clinical bloodstream infection we added the presence of a raised serial CRP as a third requirement (At an interval >12 hours following initial blood sample at onset of sepsis, CRP >10 mg/L or CRP further increased compared to initial sample if elevated CRP present at sepsis onset). Late-onset neonatal sepsis was defined as neonatal sepsis with onset > 48 hours after birth. The control group consisted of sixteen VLBW infants without sepsis admitted to the NICU during the same period.

#### Meningococcal sepsis sub study

In the meningococcal sepsis group we included twenty-eight patients with meningococcal septic shock of whom stored blood samples were available. All patients were enrolled in a clinical trial of activated protein C between July 1997 and February 2000 at the Pediatric Intensive Care Unit (PICU) of the Sophia Children’s Hospital, Erasmus Medical Centre [14]. The number of cases in the meningococcal sepsis group was determined by number of patients of whom of stored frozen serum samples were available (28 of 40). Meningococcal sepsis was defined as described previously in accordance with the recommendations of the international Pediatric Sepsis Consensus Conference, *Neisseria* *meningitidis* was isolated from blood in 26/28 (92%) [14,15]. In the control group for the meningococcal sepsis study we included sixteen patients, who had presented at the emergency room with fever for whom a diagnosis of meningitis was excluded by lumbar puncture and of whom stored blood samples were available. All control patients were enrolled between January 1998 and January 2000 at the University Medical center Utrecht [16]. The number of controls in the meningococcal sepsis control group was determined by number of patients of whom of stored frozen serum samples were available (16 of 18). For all children in the control group severe bacterial infection was ruled out, and all were diagnosed tentatively as “viral illness”.

#### Clinical and laboratory data

Clinical characteristics, disease severity scores (Score for Neonatal Acute Physiology (SNAP) II, Pediatric Risk of Mortality (PRISM) III), and laboratory parameters including CRP at sepsis onset and peak CRP (maximal CRP values during the same sepsis episode) were collected from medical records and a computerized patient data information system at study entry and during the course of the disease. Serum CRP levels were measured by a nephelometric assay, normal levels are less than 10 mg/L. Patients were monitored for 28 days or until death or hospital discharge.

### Sample collection and CEACAM1 measurement

#### Neonatal sepsis sub study

In late-onset neonatal sepsis patients blood was collected in lithium heparine or EDTA tubes median at 32 hours (range 20-122 hours; in 9 patients at 20-36h) after sepsis onset (lithium heparine samples in 3/12 infants with sepsis versus 2/16 controls). We determined CEACAM1 expression on the CD4^+^ T cells by flow cytometry. Before the start of the study it was established that measurement in lithium heparin or EDTA samples did not result in different CEACAM1 expression values (data not shown). Fresh samples were analyzed, fifty microliter whole blood samples were transferred to 50 ml tubes (Falcon, BD biosciences). The red blood cells were then lysed and the cells were then labeled with the following antibodies CD3 (SK7; BD biosciences; 1:5), CD4 (SK3; BD biosciences; 1:50), and CD66a/CEACAM1 (283340; R&D Systems; 10μL to 100,000 cells) at for 20 minutes in the dark on ice. After being washed 3 times the cells were fixed with 0.1% paraformaldehyde and analyzed on a BD four-color flow cytometer (BD Bio-sciences) and analyzed with Cell quest software. Lymphocytes were identified by CD3/CD4 labeling. A minimum of 10,000 leukocytes/sample were measured. No absolute numbers of CEACAM1 expressing CD4^+^ T cells was determined, only the percentage CD4^+^ T cells expressing CEACAM1.

#### Meningococcal sepsis sub study

In meningococcal sepsis patients serum samples were collected at ICU admission, 24-48 hours, 7-8 days and 3 months. Serum samples in the meningococcal sepsis control group were collected at the time of initial emergency room presentation with fever. Serum samples were stored at -80 ^o^C. Soluble CEACAM1 in serum was determined by a commercially available immunoassay (DY2244, R&D Systems) and performed according to the manufacturer’s specifications. In addition to determining the absolute concentration of soluble CEACAM1 in serum we also calculated the magnitude of change in sequential samples of individual patients at different time points when compared to serum soluble CEACAM1 concentrations on PICU admission.

### Statistical analysis

Data are expressed as mean ± standard deviation, unless stated otherwise. Comparisons between groups were analyzed by the Students t-test or one-way ANOVA with Bonferoni correction where appropriate. Correlations were tested by Pearson’s correlation coefficients. Differences were considered statistically significant when P <0.05. Correlations of CEACAM1 expression with maximal CRP or disease severity scores were tested one-sided, all other tests were two-sided.

## Results

### Neonatal sepsis sub study

**Figure 1:**
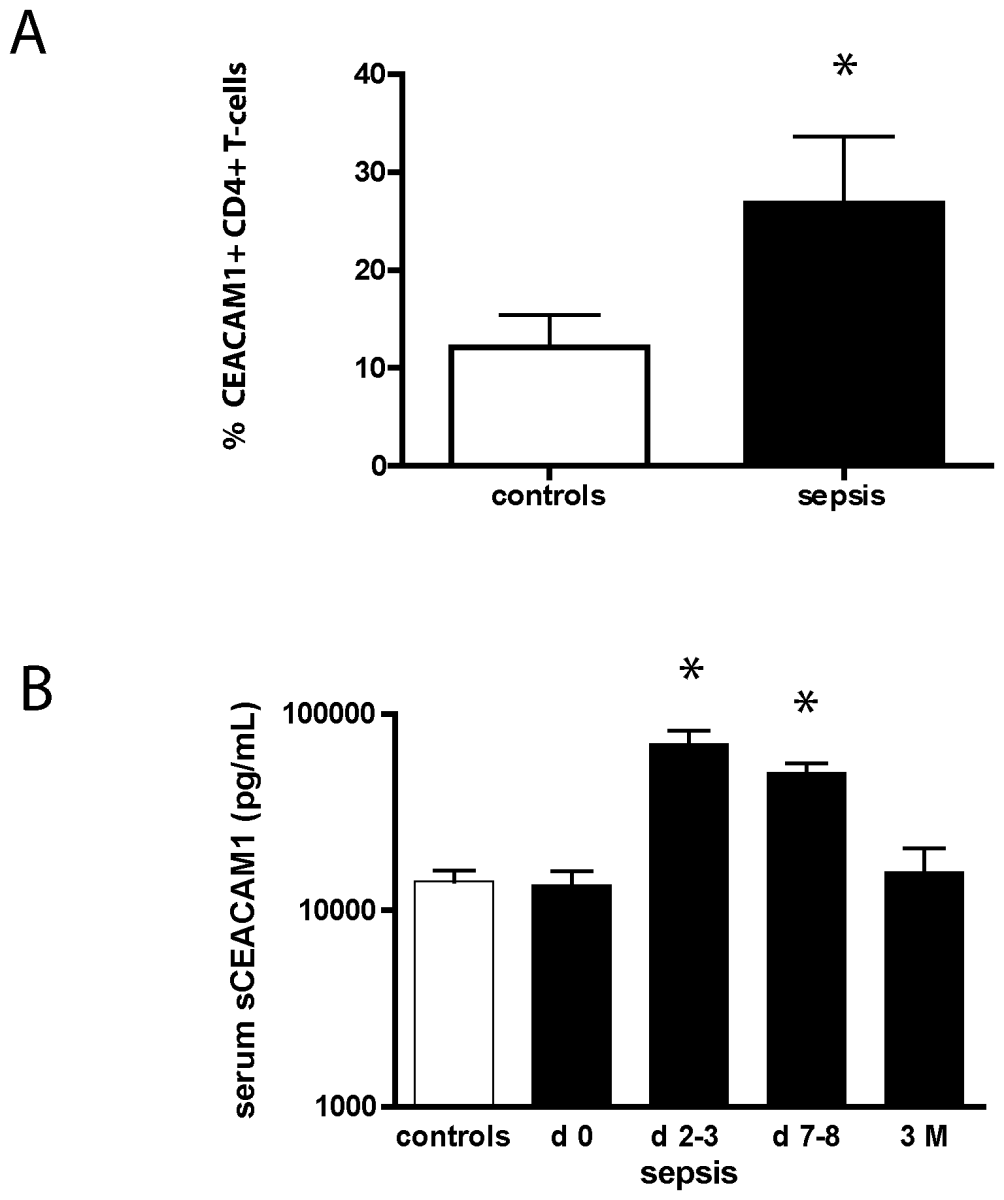
Expression of Carcinoembryonic antigen-related cell-adhesion molecule 1 (CEACAM1) in sepsis. **A**. Percentage CEACAM1 positive CD4+ T cells as determined by flow cytometric analysis in very low birth weight infants with late-onset neonatal sepsis (n=12) and non-septic controls (n=16). Data were analyzed by the Students t-test. Asterix indicates P<0.05. **B** Concentrations of serum soluble CEACAM1 in children with meningococcal septic shock (T_0h_ n=28; T_24-48h_ n=20; T_7-8d_ n=11; T_3M_ n=6) compared to healthy controls (n=16). Concentrations of soluble CEACAM1 were measured by ELISA. Levels were higher in children with septic shock at 24-48h and elevated levels persisted at day 7-8. Differences were analyzed by one-way ANOVA with Bonferoni correction. Asterix indicates P<0.05. Data are expressed as mean ± standard error.

Characteristics of the patients and controls are shown in Table 1. The percentage CEACAM1 positive CD4+ T-cells in the late-onset sepsis group were higher than in the VLBW infant control group (26.8% ± 24.0 versus 12.0% ± 13.0; P=0.046; see Figure 1A).

**Table 1:** Characteristics of patients and controls.

**late-onset neonatal sepsis substudy**	**sepsis patients**	**controls**
	(**n=12**)	(**n=16**)
Male/female ratio	6/6	10/6
Birthweight (g)	1258 (650-1465)	955 (640-1465)
Gestational age at birth (weeks)	28.9 (26.4-31.0)	27.3 (25.1-33.3)
Gestational age at sepsis onset/blood sampling (w)	30.6 (27.6-36.9)	28.4 (26.6-36.1)
SNAP II at sepsis onset/blood sampling	4 (0-32)	3 (0-29)
CRP at sepsis onset (mg/L)	15 (1-123)	1 (1-6)
Peak CRP (mg/L)	61 (1-228)	2 (1-9)
**meningococcal sepsis substudy**	**sepsis patients**	**controls**
	(**n=28**)	**(n=16)**
Male/female ratio	16/12	12/4
Age (years)	2.3 (0.3-16)	1.0 (0.1-14)
PRISM III score	24 ± 9		
CRP at sepsis onset (mg/L)	76 ± 48		
Peak CRP (mg/L)	174 ± 92		

Male/female ratio data are numbers of patients; birthweight and age data are median (range); all other data are mean ± standard deviation

**Figure 2:**
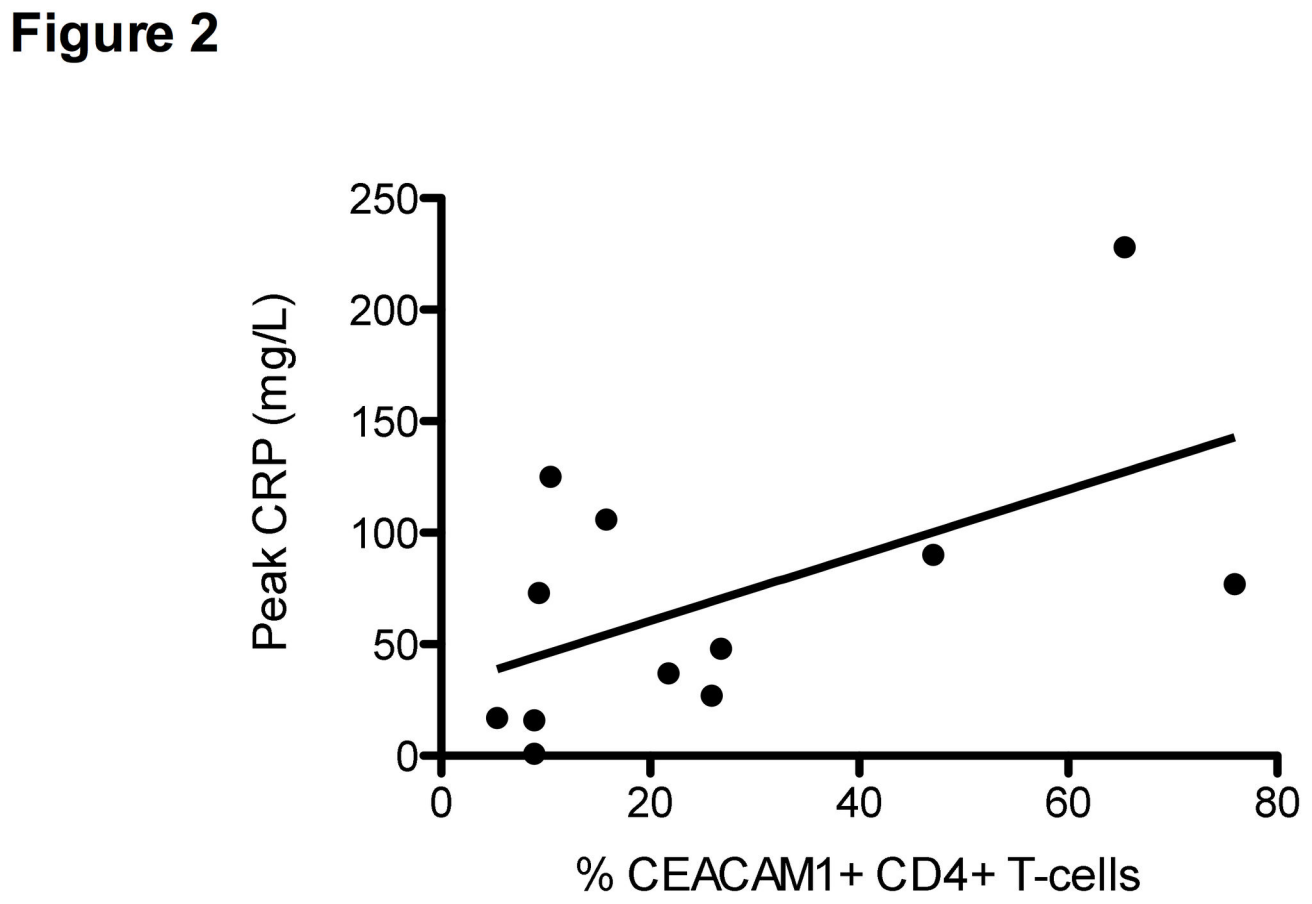
Percentage CEACAM1 positive CD4+ T-cells correlate with Peak CRP. Data represent Peak CRP levels during the course of sepsis and Percentage CEACAM1 positive CD4+ T-cells at a median of 32 hours (range 20-122 hours) after sepsis onset in late-onset neonatal sepsis in VLBM-infants. Linear regression line is drawn. Higher Peak CRP levels correlate with higher percentage CEACAM1 positive CD4+ T-cells (Pearson’s correlation coefficient R= 0.553; P < 0.05).

Sepsis associated mortality was 1/12 (8.3%). The percentage CEACAM1 positive CD4+ T-cells in VLBW infants was not related to gestational age, antenatal steroids, and time of sampling after sepsis onset. The peak CRP level 70 mg/L ± 63 as a measure of acute phase response and inflammation showed a moderate but significant correlation with percentage CEACAM1 positive CD4+ T-cells in sepsis patients (R=0.553, P =0.031; see Figure 2).

### Meningococcal sepsis sub study

Characteristics of the patients and controls are shown in Table 1. Serum soluble CEACAM1 concentrations were not elevated in meningococcal septic shock patients at PICU admission.

Serum soluble CEACAM1 concentrations at 24-48 hours were significantly elevated compared to controls and values at ICU admission respectively (70.4 ng/mL ± 52.6 versus 14.0 ng/mL ± 7.9 versus 13.5 ng/mL ± 12,9; P < 0.05; see Figure 1B).

Soluble CEACAM1 levels were not associated with administration of the trial drug activated protein C (data not shown). Serum soluble CEACAM1 concentrations were not correlated with disease severity measured by PRISM score or peak CRP.

Septic shock associated mortality was 8/28 (27%), Seven deaths occurred within the first 24 hours following ICU admission, one death within 48 hours following ICU admission. Soluble CEACAM1 levels on ICU admission were not different for survivors and non-survivors (14.4 ng/mL ± 15.0 versus 10.9 ng/mL ± 4.1; P= 0.16), and the 24-48h values in survivors were comparable to the 24 hour serum concentration of soluble CEACAM1 in the single patient that died after the first 24 hours following ICU admission (70.6 ng/mL ± 54.0 versus 65.6 ng/mL).

Soluble CEACAM1 levels were persistently elevated at day 7-8 (50.2 ng/mL ± 12.9; P < 0.05), but had returned to normal values in convalescent serum samples drawn 3 months after sepsis onset (15.7 ng/mL ± 12.3; see Figure 1B). Analysis of the magnitude of change of serum soluble CEACAM1 concentrations in comparison to individual PICU admission values was consistent with analysis of absolute values (24-48h: 569% ± 173; P < 0.05; day 7-8: 483% ± 253; P< 0.05; 3 months: 159% ± 170; P=0.2).

## Discussion

The primary finding in this study suggests that late-onset neonatal sepsis in VLBW-infants causes an increase in the percentage circulating CD4+ T-cells expressing CEACAM1. In addition, our data show meningococcal septic shock is associated with a significant and persistent increase in circulating soluble CEACAM1 concentration from 24–48hours up to day 7-8 following PICU admittance.

In the VLBW infants with late-onset neonatal sepsis CEACAM1 expression on the CD4+ T-cells correlated with the maximal CRP levels, while in children with meningococcal septic shock serum soluble CEACAM1 concentrations did not correlate with CRP. In the present study we did not assess the absolute numbers of CD4+ T-cells, thus we cannot determine whether the observed increase is relative or absolute. Effect of treatment in the ICU (e.g. antibiotics, vasopressive drugs) on CEACAM1 levels cannot be excluded from our study. No correlation between the percentage CEACAM1 positive CD4+ T-cells or levels of soluble CEACAM1 and clinical disease severity scores was demonstrated. Our study was limited in size and larger studies to confirm our findings also in different age groups and in patients with different sepsis etiologies are warranted.

The CEACAM1 molecule in humans displays considerable variation, 11 different CEACAM1 splice variants have been detected. Splice variants differ in the number of extracellular immunoglobulin-like domains, membrane anchorage, and also the length of their cytoplasmic tails. Splice variants in transmembrane and intracellular domains have functional significance. Isotypes with short cytoplasmic tails lack inhibitory function. Regulation of expression of different isotypes can vary with cellular activation state. In general long cytoplasmic tail isotypes are more abundant and CEACAM1 is generally seen as an inhibitory immune co-receptor [9].

Not surface expressed, but soluble isotypes of CEACAM1 also mediate biological functions, by activation of surface expressed CEACAM1, or by interference with binding of CEACAM1 to other surface expressed CEACAM1 molecules.

In the present study we did not address the variation introduced by CEACAM1 splice variants. It will be valuable to assess in future studies and to assess the relative expression of functionally different CEACAM1 isoforms.

Consistent with findings in human adults, CEACAM1 was expressed on a low percentage human peripheral-blood CD4+ T-cells in non-septic VLBW-infants [9]. Certain pathologic conditions (coeliac disease and inflammatory bowel disease) have previously been shown to cause increased CEACAM1 expression on T cells in the lamina propria of the gut [12,17]. In vitro activation of T-cells by cytokines such as IL-2, IL-7 and IL-15 causes rapid and strong CEACAM1 up regulation, which persists for many days. At present there is debate on the role of these cytokines in sepsis in vivo, and the potential mechanism by which these cytokines may prevent immune dysfunction (Please see also comment on reference 18 and 19 by E.D. Carrol J Immunol 2010) [18,19]. [12,17]

We are the first to demonstrate an increase in CEACAM1 positive CD4+ T-cells in peripheral blood in vivo in humans in sepsis. Since CEACAM1 generally functions as an inhibitor of T-cell receptor activation, increased CD4+ T-cells CEACAM1 expression in sepsis may contribute to the suppression of T cell functions as observed in sepsis. Soluble CEACAM1 may function as a ligand for CEACAM1 and thus altered concentrations of soluble CEACAM1 in sepsis may further influence T-cell functions. Furthermore CEACAM1 is also expressed on innate immune cells, such as neutrophils, monocytes, and natutal killer cells, and altered soluble CEACAM1 concentrations in sepsis may directly influence neutrophil and monocyte survival [20,21]. In addition soluble CEACAM1 may interfere with CEACAM1 mediated cell-cell contact and thus influence immune regulation, as demonstrated for natural killer cells [22].

CEACAM1 is also reported to inhibit Toll-like Receptor-2 signaling and Toll-like Receptor-4, thus increased circulating soluble CEACAM1 might contribute to inhibition of Toll-like Receptor responses in sepsis [23,24].

Interestingly, in genetically engineered mice that are resistant to apoptosis due to transfection with the anti-apoptosis gene Bcl-2, sepsis results in uniquely decreased transcription of CEACAM1 in splenocytes and increased sepsis survival [25]. Whether a decrease in splenocyte CEACAM1 expression improves sepsis survival, or whether there is no causal relation, is unknown.

From our data we can not exclude that the increase in percentage CEACAM1 positive CD4+ T-cells is caused by a greater loss of CEACAM1 negative CD4+ T-cells for instance by apoptosis.

It will be valuable to determine whether sepsis causes a relative or absolute increase in CEACAM1 expressing CD4+ T-cells in future studies. Further determination of the functional role of CEACAM1 in sepsis seems justified, as targeting CEACAM1 might be of potential therapeutic benefit in sepsis.

Pathogens including *Neisseria meningitides* also bind CEACAM1 and current data on immune modulating effects of such interactions are conflicting [9,26,27]. Thus circulating soluble CEACAM1 in children with meningococcal sepsis may also bind whole bacterial cells or blebs in the circulation and might further affect the immune response to meningococci. Further research will be needed to evaluate the effects of such interactions on the immune response and overall course of disease.

In conclusion our data demonstrate increased surface expression of the co-inhibitory immune receptor CEACAM1 in late-onset neonatal sepsis in VLBW-infants, and increased circulating CEACAM1 self-ligand soluble CEACAM1 in children with meningococcal sepsis. Increased T-cell CEACAM1 expression and increased circulating soluble CEACAM1 may contribute to sepsis-associated immune suppression.
